# Fully Automatic Treatment Planning for External-Beam Radiation Therapy of Locally Advanced Cervical Cancer: A Tool for Low-Resource Clinics

**DOI:** 10.1200/JGO.18.00107

**Published:** 2019-01-10

**Authors:** Kelly Kisling, Lifei Zhang, Hannah Simonds, Nazia Fakie, Jinzhong Yang, Rachel McCarroll, Peter Balter, Hester Burger, Oliver Bogler, Rebecca Howell, Kathleen Schmeler, Mike Mejia, Beth M. Beadle, Anuja Jhingran, Laurence Court

**Affiliations:** **Kelly Kisling**, **Lifei Zhang**, **Jinzhong Yang**, **Rachel McCarroll**, **Peter Balter**, **Rebecca Howell**, **Kathleen Schmeler**, **Anuja Jhingran**, and **Laurence Court**, The University of Texas MD Anderson Cancer Center, Houston, TX; **Hannah Simonds**, Stellenbosch University and Tygerberg Hospital; **Nazia Fakie** and **Hester Burger**, University of Cape Town and Groote Schuur Hospital, Cape Town, South Africa; **Oliver Bogler**, The University of New Mexico School of Medicine, Albuquerque, NM; **Mike Mejia**, University of Santo Tomas Hospital, Benavides Cancer Institute, Manila, Philippines; **Beth M. Beadle**, Stanford University, Stanford, CA

## Abstract

**Purpose:**

The purpose of this study was to validate a fully automatic treatment planning system for conventional radiotherapy of cervical cancer. This system was developed to mitigate staff shortages in low-resource clinics.

**Methods:**

In collaboration with hospitals in South Africa and the United States, we have developed the Radiation Planning Assistant (RPA), which includes algorithms for automating every step of planning: delineating the body contour, detecting the marked isocenter, designing the treatment-beam apertures, and optimizing the beam weights to minimize dose heterogeneity. First, we validated the RPA retrospectively on 150 planning computed tomography (CT) scans. We then tested it remotely on 14 planning CT scans at two South African hospitals. Finally, automatically planned treatment beams were clinically deployed at our institution.

**Results:**

The automatically and manually delineated body contours agreed well (median mean surface distance, 0.6 mm; range, 0.4 to 1.9 mm). The automatically and manually detected marked isocenters agreed well (mean difference, 1.1 mm; range, 0.1 to 2.9 mm). In validating the automatically designed beam apertures, two physicians, one from our institution and one from a South African partner institution, rated 91% and 88% of plans acceptable for treatment, respectively. The use of automatically optimized beam weights reduced the maximum dose significantly (median, −1.9%; *P* < .001). Of the 14 plans from South Africa, 100% were rated clinically acceptable. Automatically planned treatment beams have been used for 24 patients with cervical cancer by physicians at our institution, with edits as needed, and its use is ongoing.

**Conclusion:**

We found that fully automatic treatment planning is effective for cervical cancer radiotherapy and may provide a reliable option for low-resource clinics. Prospective studies are ongoing in the United States and are planned with partner clinics.

## INTRODUCTION

Global cancer rates are increasing, especially in low- and middle-income countries.^1^ By 2025, 20 million cancer cases are predicted worldwide annually,^2^ of which half would benefit from treatment with radiation therapy.^3-5^ However, many countries lack adequate radiation therapy capabilities^5^; this is due, in part, to staff shortages in these regions.^6^ Automating radiation treatment planning could help mitigate this limitation by allowing technology to do a large part of the required work to begin treatment of patients. In addition, an expedited planning process could enable patients to be treated much sooner after diagnosis.

**Fig 1:**
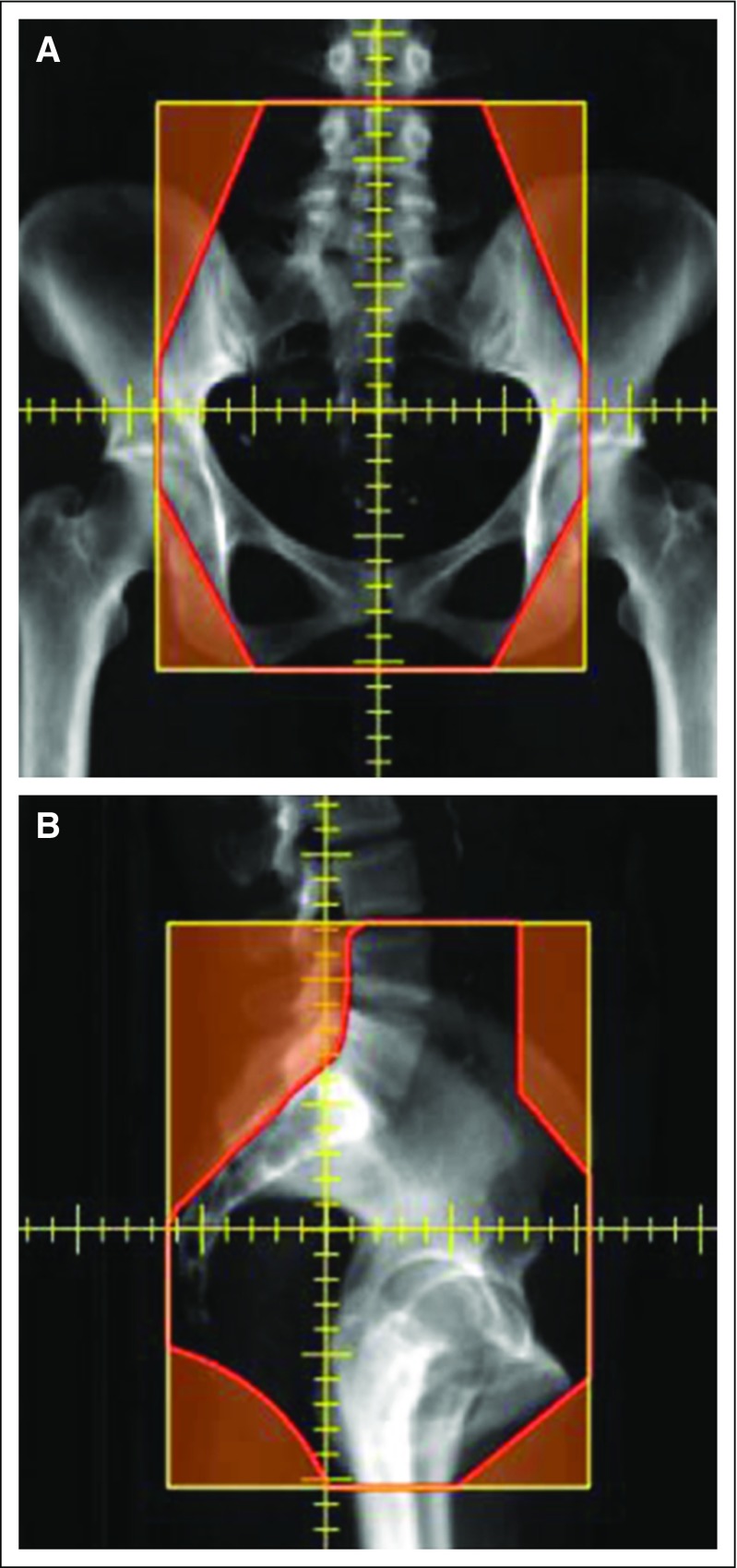
Automatically created treatment fields. Beam’s eye view of the (A) anteroposterior (AP) and (B) right lateral beam angles. The beam apertures are designed on the basis of the bony anatomy and will be collimated using the multileaf collimator.

According to ASCO and the International Atomic Energy Agency, the recommended treatment of invasive cervical cancer in low-resource settings is a radiotherapy technique known as a “four-field box.”^7,8^ This technique uses four orthogonal beams to treat the gross tumor and at-risk tissues in the pelvis. The beam apertures are based on bony anatomy that is visible in a digitally reconstructed radiograph from each of the beam angles: anteroposterior, posteroanterior, right lateral, and left lateral. Examples of beam apertures are shown in Figure 1.

Through a collaboration with hospitals in South Africa and the United States, we have developed a fully automatic treatment planning tool, the Radiation Planning Assistant (RPA).^9^ The RPA designs patient-specific four-field box radiation treatments for locally advanced cervical cancer, one of the most prevalent forms of cancer in low-resource settings.^1^ To build the RPA, we developed algorithms to automate every step in the treatment planning process. The RPA has been integrated with a commercial treatment planning system (TPS) to plan three-dimensional treatments on planning CT scans with no human input.

The objective of this study was to validate the individual algorithms of the RPA and to test the fully integrated system on patient CT scans. We retrospectively tested the RPA using patient CT scans from cancer hospitals in the United States and in South Africa. We have also implemented a semiautomated version of the RPA into the clinical workflow at The University of Texas MD Anderson Cancer Center (Houston, TX; hereafter, MD Anderson).

## METHODS

All studies and patient data were handled in accordance with the corresponding approved institutional review board protocol, and where required, patient consent was obtained.

### Overview of the RPA

To plan a patient-specific treatment with the RPA, the following inputs are used: (1) a CT scan of the patient in the treatment position and (2) a plan order from the physician, which includes basic patient information, including the prescription. With no further human input, the RPA automatically creates a treatment plan that is ready for physician review, along with plan documentation. This documentation is for the patient’s medical record and for performing quality assurance checks that are vital to delivering safe radiotherapy.^10^ The documentation includes all dose distributions, allowing the physician to review the quality of the plan, including target coverage.

Algorithms have been developed to automate each manual step of treatment planning and have been integrated with the Eclipse TPS (Varian Medical Systems, Palo Alto, CA) using its Application Programming Interface to form the fully automatic TPS. The algorithms that automate each step are described in the following section.

### In-House Automation Algorithms

#### Delineation of the body contour.

**Fig 2:**
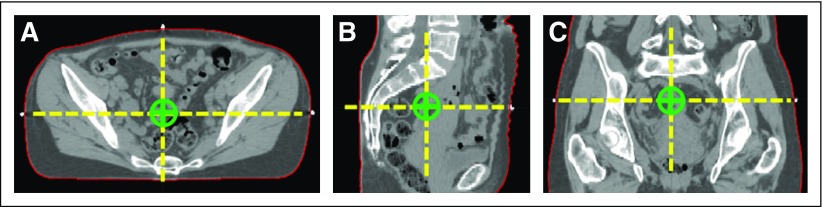
Body contour and marked isocenter. (A-C) Three views, (A) axial, (B) sagittal, and (C) coronal, of the computed tomography scan of a patient. The automatically segmented body contour is outlined in red. The views intersect at the location of the marked isocenter (green), which is determined on the basis of the radiopaque external fiducials. The intersecting planes are denoted by the dashed yellow line.

The body contour (Fig 2) is important for accurate dose calculation in the Eclipse TPS. The first step in this algorithm is to identify the location of the couch using the sum projection signal along the lateral direction and then searching for the most representative peak. The couch is then removed from the image by setting all pixels posterior to this line to the CT number of air. The RPA then searches for the body contour by thresholding the CT image intensity (with the couch removed) into a binary mask; it then uses postprocessing to ensure the topologic characteristics and smoothness.

#### Detection of the marked isocenter.

The next step in the RPA is to automatically detect the marked isocenter, as indicated by the intersection of three radiopaque fiducials placed on the patient’s skin during the planning CT scan (Fig 2). The RPA automatically detects the marked isocenter by defining a search domain within the bandwidth of the body contour. Potential fiducial candidates within the search domain are identified on the basis of the CT number. Any false candidates are removed using several criteria, including size, location, and geometry. Finally, the intersection of the selected cluster of three fiducials is used to define the marked isocenter.

#### Design of the treatment field apertures.

**Fig 3:**
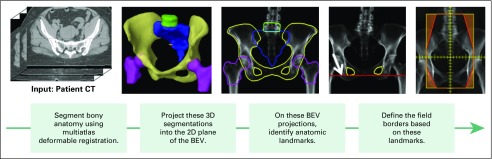
Workflow of the algorithm that automatically designs four-field box treatment beams. For automated planning, the only input is a computed tomography scan and a prescription. No other human input is required, and a plan is presented for physician review. 2D, two dimensional; 3D, three dimensional; BEV, beam’s eye view

The RPA then automatically designs the four orthogonal treatment beams, which intersect at the marked isocenter. First, the RPA automatically segments the following bony anatomy on the CT image: bony pelvis, femoral heads, sacrum, and fourth and fifth lumbar vertebral bodies. The RPA uses a deformable, multiatlas technique for automatic segmentation.^11,12^ Next, the RPA projects the segmented anatomy into each beam’s eye view (BEV). The RPA automatically identifies anatomic landmarks in the BEV, such as the widest extent of the pelvic inlet, and sets the beam aperture on the basis of these landmarks according to a set of defined rules (eg, 2 cm wider than the pelvic inlet). A representation of this workflow is shown in Figure 3.

#### Optimization of the dose distribution.

Next, the RPA creates the treatment beams in the Eclipse TPS using the automatically defined beam apertures set at the automatically located isocenter. The RPA then automatically calculates the dose delivered by each beam using 18-MV photons. To achieve a homogenous dose distribution within the treated volume, the RPA automatically determines the weighted contribution of each beam. The RPA uses a least-squares fitting to determine the beam weights that minimize the dose heterogeneity inside the treated volume. The treated volume is defined as the volume intersected by all beams, contracted by a 0.5-cm margin to exclude the rapid dose drop-off at the field edge.

### Retrospective Testing of RPA Algorithms

We first tested each algorithm retrospectively on 150 pelvic CT scans of female patients at MD Anderson. Then we tested the fully integrated RPA on 10 CT scans of female patients from Tygerberg Hospital (Cape Town, South Africa) and four CT scans from Groote Schuur Hospital (Cape Town, South Africa). All CT scans had been acquired for radiotherapy planning, with the patients supine.

#### Delineation of the body contour.

The automatically delineated body contour from the RPA was compared with the body contour resulting from Eclipse’s semiautomated body contour tool, with manual edits where necessary. The two body contours were compared quantitatively using the Dice similarity coefficient, mean surface distance, and Hausdorff distance.^11^

#### Detection of the marked isocenter.

The automatically localized marked isocenter was compared with an isocenter that had been manually placed at the intersection of the three fiducial markers. The absolute distance between these two points was calculated and used for comparison.

#### Design of the treatment-field apertures.

The automatically created treatment-field apertures were reviewed by two physicians specializing in gynecologic radiation oncology, one from MD Anderson (A.J.) and one from Tygerberg Hospital in South Africa (H.S.). They rated each field as “acceptable” or “not acceptable” for treatment, on the basis of whether they would treat the patient using that field. For a plan to be acceptable, all four fields must have been rated as acceptable.

#### Optimization of the dose distribution.

The dose distributions were calculated using automatically optimized beam weights and were compared with nonoptimized dose distributions, which used equally weighted beams (ie, each beam contributed the same dose to the calculation point). The maximum dose, defined by the hottest 1 cc of tissue, was evaluated. We also assessed the coverage, defined by the percentage of the treated volume covered by at least 95% of the prescription dose. The values with and without automated beam weight optimization were compared using a Wilcoxon signed-rank test.

#### Running time of the RPA system.

The time for the RPA to automatically plan a treatment was recorded. This included every step, beginning from the import of the CT scan and plan order into the RPA and ending with the optimized and calculated treatment plan in the TPS, ready for physician review.

#### Running the RPA remotely on patients at two South African hospitals.

The fully integrated RPA was tested on-site at Tygerberg Hospital and Groote Schuur Hospital. The resulting treatment plans and dose distributions were reviewed by physicians specializing in gynecologic oncology at the corresponding hospital (H.S., N.F.) and rated as acceptable or not acceptable for treatment.

### Clinical Deployment at MD Anderson

A semiautomated version of the RPA was created and deployed into the clinical workflow at MD Anderson for patients with cervical cancer in July 2016. This version was integrated with the Pinnacle TPS (Philips Healthcare, Andover, MA). The workflow of this system differs from the fully automated workflow in that the physician manually contours the soft-tissue target volumes on the CT scan. After the CT scan is imported into the TPS, the dosimetrist exports the CT scan to the RPA, The RPA then automatically detects the marked isocenter and designs the treatment-field apertures (still based on the bony anatomy). Once complete, the RPA automatically sends an e-mail indicating that the plan is ready, and the dosimetrist imports the uncalculated treatment beams. The physician reviews the beams, making any necessary edits on the basis of the contours of the target and critical structures, and planning continues.

We assessed any manual changes to the location of the marked isocenter. We also quantitatively compared the extent of the physician edits to the automatically planned beam apertures, using the mean surface distance and Hausdorff distance.

## RESULTS

### Retrospective Testing of RPA Algorithms

#### Delineation of the body contour.

A typical result of the automatically delineated body contour is shown in Figure 2. This body contour agreed well with the contour generated using Eclipse’s semiautomatic tool with manual edits. The median Dice similarity coefficient was 0.996 (standard deviation [SD], 0.001; range, 0.988 to 0.997). The median mean surface distance was 0.6 (SD, 0.2; range, 0.4 to 1.9) mm. The median Hausdorff distance was 22.3 (SD, 18.6; range, 5.7 to 122.7) mm.

The largest discrepancies were found when the patient’s arm was included in only one of the contours. Although these differences may seem large in some patients, they result from differences in how each technique handled the inclusion of the patients’ arms. These discrepancies are outside the treatment area and would not affect the dose delivered.

#### Detection of the marked isocenter.

The distances between automatically and manually placed marked isocenters were small (average, 1.1 mm; SD, 0.7; range, 0.1 to 2.9 mm). The largest discrepancies were found when the fiducials did not all appear on the same axial slice of the patient’s CT scan. This sometimes led to the isocenters being located on adjacent slices.

#### Design of the treatment-field apertures.

An example of the treatment fields generated by the RPA are shown in Figure 1. Of the 150 treatment plans (n = 600 fields), one physician rated 136 (91%) as acceptable for treatment. The second physician found that the image quality of the BEV was too poor in six of 150 plans (four had large amounts of bowel contrast that partially obstructed the bony anatomy in the BEV) and did not rate these six plans. Of the remaining 144 plans, the physician rated 126 (88%) as acceptable. Of the plans marked as unacceptable by at least one physician (n = 23), 19 (83%) had incorrectly placed superior borders as a result of inaccurate contouring of the vertebral bodies during the automatic segmentation step. To overcome this, we will incorporate an option to manually adjust this border in the workspace of the RPA where the physician reviews the treatment plan.

#### Optimization of the dose distribution.

**Fig 4:**
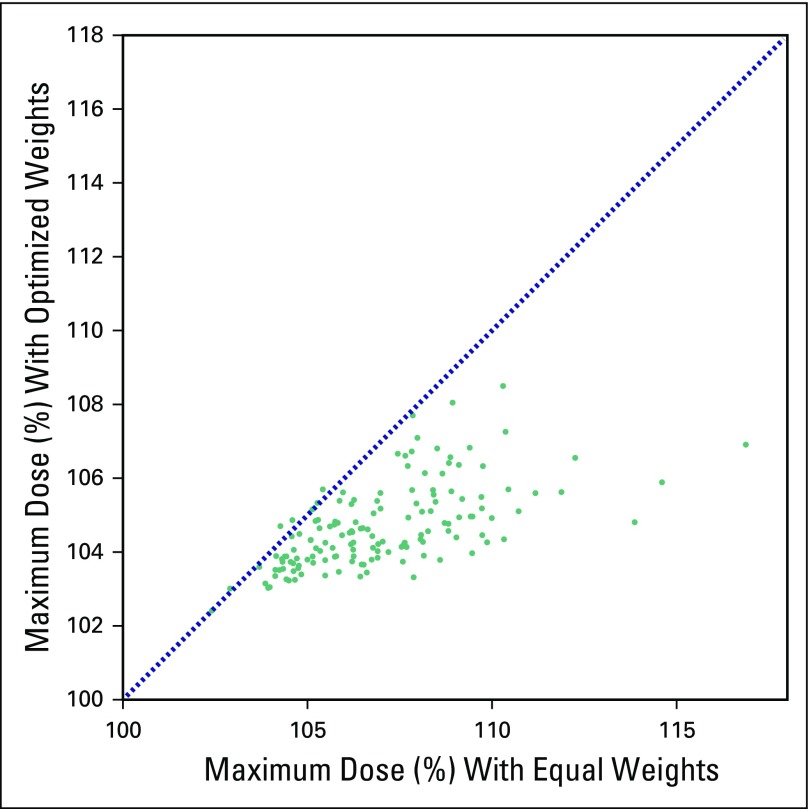
Maximum dose was reduced using automatic beam-weight optimization. The maximum dose (hottest 1 cc) is shown for each patient (n = 149) as a percentage of the prescription dose for optimized versus equal beam weights (nonoptimized). The dotted line represents no change in the maximum dose, and all points below this line showed a reduction in the maximum dose. The reduction was especially large for patients who had very high maximum doses using equal beam weights.

Figure 4 shows a comparison of the maximum dose for each patient with automatic beam-weight optimization versus without optimization. The maximum dose was significantly lower using automatically optimized beam weights, with a median change of −1.9% (*P* < 0.001, range −10.0% to +0.4%). In addition, there was a small yet statistically significant increase in the coverage of the treated volume. The median percentage of the volume covered by 95% of the prescription increased by 0.6% (*P* < .001, range: −2.8% to +2.8%).

**Fig 5:**
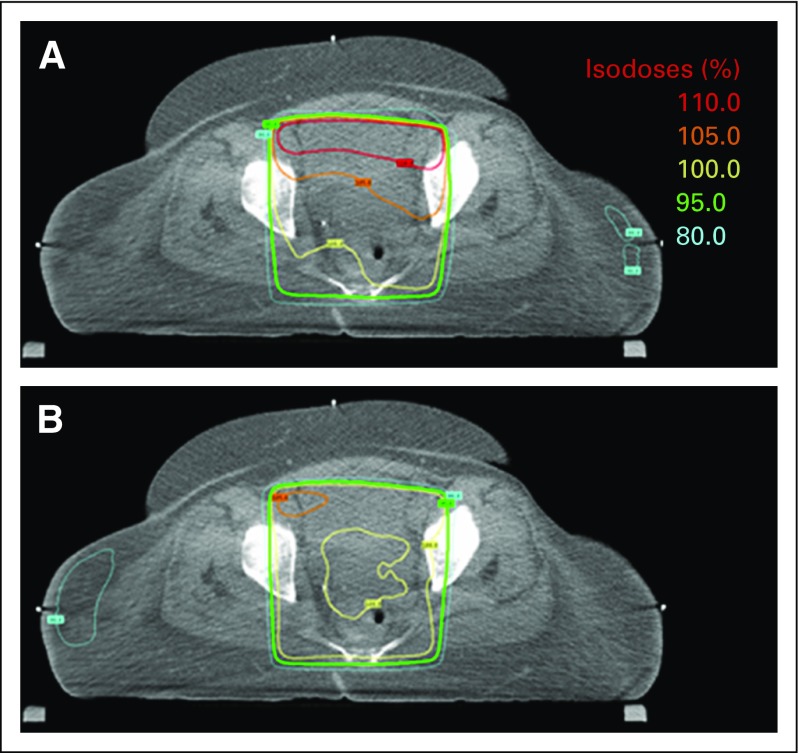
Patient plans with high maximum doses experience a substantial reduction in the maximum dose with automatic beam-weight optimization. The resulting dose distribution for an (A) automatically planned four-field box with equal beam weights (nonoptimized) and (B) automatically optimized beam weights. The maximum dose was reduced from 117% to 107% of the prescription dose for this patient.

The use of automatic beam-weight optimization was especially beneficial for patients with high maximum dose (≥ 107% of the prescription dose) without optimization. These patients’ plans experienced a larger median change in maximum dose (−3.5%). Furthermore, the percentage of patients with high maximum doses was reduced from 44% without optimization to 3% with optimization. Figure 5 shows the dose distribution of one axial slice from one patient whose very high maximum dose was greatly reduced using optimized beam weights.

#### Running time of the RPA system.

Once the planning CT scan and plan order were imported, the fully integrated RPA created a plan in Eclipse ready for physician review in a median of 11.0 minutes (range, 8.2 to 13.6 minutes).

#### Running the RPA remotely in patients at two South African hospitals.

Of the 14 treatment plans created on the planning CT scans of patients from the partner hospitals in South Africa, 100% were approved for treatment by the physician (10 plans from Tygerberg Hospital and four from Groote Schuur Hospital).

### Clinical Deployment at MD Anderson

Since the clinical version of the RPA was deployed at MD Anderson, it has been used in the planning of 24 patients with cervical cancer. The location of the marked isocenter was not adjusted for 20 patients and was adjusted less than 1 mm for four patients. The physicians edited the automatically created treatment fields on the basis of their contours of the target and normal tissues. When comparing the fields before and after physician edits, the median mean surface distance was 3.5 mm (SD, 2.4 mm; range, 0.0 to 10.4 mm) and the median Hausdorff distance was 13.9 (SD, 9.1; range, 0.0 to 42.0) mm.

## DISCUSSION

In this work, we validated the RPA’s algorithms with physician review of a large cohort of patients and performed remote testing of the fully integrated RPA. This work represents a critical step before implementation of the fully automated system in the clinic. To our knowledge, this is the first work toward automated treatment planning for radiation therapy of cervical cancer.

Before this study, the algorithms for defining the beam apertures were honed over several testing iterations on more than 250 patient CT scans with feedback from physicians at MD Anderson and Tygerberg Hospital and on the basis of the clinical edits made by physicians using the MD Anderson–deployed version of the RPA. The final algorithm, validated in this study, was a consensus of the radiation oncologists for patients whose disease extent was limited to the upper two-thirds of the vagina and with only pelvic lymph node involvement. In the future, we can extend this work for patients with more advanced disease (eg, involvement of the distal vagina or paraaortic nodes) by including variations on the beam-aperture definitions. Within the RPA workflow, the rules by which the beam apertures are defined can be adjusted for a range of disease stages, as long as these rules are based on automatically segmented bony anatomy.

In addition to extensive retrospective testing at MD Anderson, we conducted a successful retrospective test of remote, fully automatic treatment planning at two clinics in South Africa. Moving forward, we will prepare for clinical deployment and testing, beginning with our two partner clinics in South Africa. We will monitor the prospective use of the RPA and evaluate its effect on clinical workflow, including the time staff spend planning and the time from CT simulation to first treatment. During this testing, we expect to address challenges on the basis of differences in clinical workflow and software and hardware platforms. Ultimately, our goal is to deploy in clinics with fewer resources, which will likely introduce new challenges in terms of staffing, workflow, and equipment. We also are evaluating options to make this tool accessible to low-resource clinics, considering that there may be limited financial resources available. In addition, we are developing automated treatment planning for head-and-neck cancer radiation therapy^13^ and postmastectomy chest-wall radiation therapy.^9^

The treatment technique planned by the RPA is recommended for cervical cancer in low-resource clinics, according to the International Atomic Energy Agency and ASCO.^7,8^ Although treatment apertures on the basis of soft-tissue contours would be preferable for curative treatments, the bony anatomy approach is used as an alternative in low-resource settings where there is a lack of staff to complete the manual contouring necessary for more conformal treatments. With plans created by the RPA, the physician can use the automatically created documentation to review the dose distribution and evaluate the plan’s coverage, even without having contoured the soft-tissue disease.

Given the prevalence of cervical cancer, the fully automatic treatment planning offered by the RPA could help alleviate staff shortages in low-resource clinics. In addition, by reducing the back-and-forth handoffs between planners and physicians needed to manually plan a treatment, the automated system could prepare a plan more quickly, presenting a plan for review shortly after the CT scan is acquired. We envision the RPA facilitating same-day treatments, where a patient never has to leave the clinic between CT scan and her first treatment. In contrast, for patients with gynecologic pelvic disease in our clinical practice, the median planning time is 21 hours (interquartile range, 7 to 47 hours) from CT simulation to when the plan is ready for physician review, including handoffs and time when the plan is not being actively worked on (unpublished data). Furthermore, handoffs between staff have been identified as a weakness in radiotherapy safety, and any reduction in the number of handoffs may result in an improvement in the safety of radiation therapy.^14,15^

The results of this study indicate that fully automatic treatment planning for cervical cancer is achievable. More prospective studies are necessary and are ongoing in the United States and planned with our international partner clinics. By reducing the work required by trained staff, the RPA could ease the burden of creating patient-specific treatment plans in resource-constrained clinics. As a result, using the RPA to automatically plan treatments could help reduce some of the barriers to establishing radiation therapy programs.
